# Knowledge to Impact: From Conceptualizing to Mobilizing the Saskatchewan Caregiver Experience Study

**DOI:** 10.1111/jep.70291

**Published:** 2025-09-30

**Authors:** Steven Hall, Noelle Rohatinsky, June Gawdun, Leslie Macala, Jennifer White, Lorraine Holtslander, Shelley Peacock

**Affiliations:** ^1^ Faculty of Nursing University of Alberta Edmonton Alberta Canada; ^2^ College of Nursing University of Saskatchewan Saskatoon Saskatchewan Canada; ^3^ Saskatoon Council on Aging Saskatoon Saskatchewan Canada

**Keywords:** community partnerships, family caregivers, knowledge translation, older adults, public health policy

## Abstract

**Objectives:**

Given the rising number of older adults reliant on family and friend caregivers (i.e., those who provide informal care), the Saskatchewan Caregiver Experience Study aimed to examine the experiences of these caregivers in Saskatchewan and identify unmet needs. This paper describes our knowledge translation and mobilization efforts of our study's findings.

**Methods:**

Researchers partnered with the Saskatoon Council on Aging (SCOA) to conduct the study, recruiting 355 family and friend caregivers. We evaluated impacts across conceptualization, data collection and knowledge mobilization using the Knowledge Engagement Impact Assessment Toolkit to assess how effectively our study's design has the potential to impact policy and practice, which involved completing an Assessment Matrix (quantitative assessment) and Assessment Portrait (qualitative assessment). A stakeholder webinar served as the primary knowledge translation event.

**Findings:**

An Assessment Matrix revealed moderate impact scores for conceptualization and knowledge mobilization phases. However, the Assessment Portrait reflected collaboration, thorough policy alignment and outreach. Data collection and analysis scored lower. We reflected on this lower score in the Assessment Portrait as being due to fewer avenues for reciprocal engagement and capacity‐building during this stage. Policy recommendations, formed in collaboration with SCOA, were presented at the webinar and called for expanded respite care and streamlined system navigation.

**Conclusion:**

By systematically evaluating research activities, this study highlights the critical role of knowledge translation in shaping caregiver support. Findings reinforce the importance of early and ongoing stakeholder collaboration, user‐friendly dissemination methods and targeted policy action. Employing a structured framework for measuring engagement impact can guide targeted interventions, ensuring that caregiver programming, legislative reforms and improved care quality align with evolving population needs and priorities.

## 1 Background

Population ageing is driving the need for family and friend caregivers across Canada to provide informal and unpaid care to older adults [1]. Supporting these individuals is critical due to the significant physical, emotional and financial burdens they face [2]. Caregivers are defined by the Canadian Institute for Health Information [3] as ‘family members, neighbours and friends who take on an unpaid caring role to support someone with a diminishing physical ability, a debilitating cognitive condition or a chronic life‐limiting illness’ (para. 2). Furthermore, the act of caregiving has been conceptually defined by Hermanns and Mastel‐Smith [4] as:

…the process of helping another person who is unable to do for themselves in a ‘holistic’ (physically, mentally, emotionally and socially) manner. Caregiving is facilitated by certain character traits, emotional skills, knowledge, time and an emotional connection with the care recipient.

(p. 15)

Family and friend caregivers (herein referred to as ‘caregivers’) often provide essential health and social support to older adults, but they themselves are at risk of negative health outcomes and financial strain, especially if they lack adequate support [2, 5, 6]. Despite the significant role caregivers play, many do not receive adequate support, which can lead to emotional and physical difficulties [2]. Furthermore, supportive services are underutilized, highlighting the need for better access and awareness of these resources [6]. There is a growing need for public policies that provide direct and indirect support to caregivers, such as financial aid and training programmes [6, 7, 8]. Ultimately, supporting caregivers of older adults is a public health issue because it directly impacts the health, economic stability and social well‐being of both caregivers and care recipients. Addressing this issue requires comprehensive policies, increased access to supportive services and public health interventions to alleviate the burdens faced by caregivers.

*Knowledge translation* is broadly defined by the Canadian Institutes of Health Research (CIHR) as the process of taking research findings and moving them into real‐world action through synthesis, dissemination, exchange and ethically sound application of knowledge [9]. Adding to that, a *knowledge user* is someone who can apply research findings to make informed decisions about health policies, programmes or practices [9]. Knowledge user involvement in the research process can vary in depth and complexity, depending on the type of research and their specific information needs. Lastly, *knowledge mobilization* refers to the process of facilitating the use of research findings by individuals and organizations who can apply that knowledge to improve policies, practices or services [10]. Knowledge mobilization bridges the gap between research and action, aiming to maximize the impact of research on society [10].

This manuscript describes our knowledge translation and mobilization efforts in sharing the findings of the Saskatchewan Caregiver Experience Study [11], as we moved to make an impact on health and social systems in the Canadian province of Saskatchewan. Our objective was to garner support for and raise awareness of caregiver programming. We describe our use of the Knowledge Engagement Impact Assessment Toolkit [10] to assess our approach from conceptualizing the Saskatchewan Caregiver Experience Study to mobilizing our findings with our community partner, the Saskatoon Council on Aging (SCOA).

### 1.1 Saskatchewan Caregiver Experience Study

The Saskatchewan Caregiver Experience Study sought to explore the lived experiences and support priorities of caregivers across the province [11]. It was conceptualized by nurse researchers at the University of Saskatchewan (S.H., N.R., L.H., S.P.) in collaboration with staff from SCOA (J.G., L.M., J.W.), a non‐profit organization and our community partner. The study was the first of its kind and had a significant response from Saskatchewan caregivers, eager to share their experiences. A total of 355 participants provided insights through a qualitative survey distributed via Facebook and community newsletters [11, 12]. Participants were geographically diverse, encompassing caregivers from urban‐large, urban‐small/medium and rural communities, offering a comprehensive view of informal and unpaid caregiving in Saskatchewan [11]. Caregivers described their roles as emotionally and physically taxing, often characterized by exhaustion, self‐doubt and a persistent struggle to balance caregiving with personal needs [13]. A notable barrier was the difficulty in navigating complex and fragmented health and social systems. Particularly in rural areas, geographic dispersion intensified these challenges, with many caregivers providing support from a distance due to the lack of local services [13].

Despite the burdens, many caregivers identified personal growth and a sense of reward as positive elements of their roles. Participants expressed a sense of honour and fulfilment in providing care to their care recipients [14]. They valued the ability to oversee the quality of care and appreciated the emotional closeness the experience fostered [14]. However, participants highlighted several areas of unmet needs and priorities for support [8]. Among the most pressing issues identified were timely, accessible help; emotional support; professional and compassionate healthcare; and improved systems and policies [8]. Many caregivers emphasized the need for practical, on‐demand assistance, such as help with daily care tasks, as well as greater recognition and accommodation of their roles within broader health and social systems [8]. These support priorities, along with findings from the other facets of the Saskatchewan Caregiver Experience Study, were translated into policy recommendations in collaboration with our community partner, SCOA.

### 1.2 Saskatoon Council on Aging

SCOA is a community‐based, not‐for‐profit, charitable organization and a vital resource centre serving a community of adults aged 55 and over in Saskatoon, Saskatchewan and surrounding areas [15]. SCOA was founded in 1991 by a group of passionate older adults and grew out of recognizing the need for a centralized resource centre for older adults. One of SCOA's many initiatives includes the Saskatoon Caregiver Information and Support Centre (SCISC), which was started when a group of caregivers came forward and indicated their need for additional support in the community. The SCISC has been an essential programme offered by SCOA since 2000. The SCISC is committed to providing valuable information and unwavering support to community caregivers. The SCOA and SCISC have thus far been the main knowledge users of the Saskatchewan Caregiver Experience Study, using findings to target and tailor caregiver support initiatives [16].

### 1.3 Knowledge Engagement Impact Assessment Toolkit

The Knowledge Engagement Impact Assessment Toolkit is a structured, flexible framework designed to help researchers and practitioners plan, monitor and evaluate the impact of their knowledge engagement work [10]. It is composed of two core components: the Assessment Matrix and the Assessment Portrait. These elements work in tandem to capture both quantitative metrics and qualitative insights about knowledge engagement activities. Users can apply this toolkit to a single activity or product (e.g., a specific engagement event or a knowledge product), to multiple components of a research project (e.g., each research question) or to an entire initiative or portfolio [10]. The toolkit can be requested from the University of Calgary [10] website, and a training video is provided along with the toolkit materials [17].

The first of the two components is the *Assessment Matrix*, which prompts users to think about their project or activity across a series of impact indicators associated with key principles: reciprocity, externalities, access and partnership (referred to as ‘REAP’), as well as its strategic context [17]. Each indicator is scored on a five‐point scale (with higher scores representing greater impact), generating a total weighted impact score that helps classify the activity's overall impact as high, moderate or low [17]. While the core impact indicators are standardized, they can be adapted by adjusting factor weightings or by adding custom indicators developed in collaboration with community partners [17]. In addition, the matrix offers space to record engagement details, making it a useful repository of relevant data throughout a project's lifecycle [17].

Complementing the Matrix, the *Assessment Portrait* captures the ‘story’ behind the scores. It guides users to document the objectives, successes, struggles and unanticipated outcomes that have shaped their knowledge engagement efforts [17]. This qualitative reflection acknowledges that engagement work is often nonlinear and complex, providing a narrative through which lessons learned, partnership development and future opportunities can be described [17]. Together, the Assessment Matrix and Portrait create a comprehensive picture of engagement impact, offering consistent language and documentation that can be used in progress reports, needs assessments and tracking knowledge translation efforts [10]. After reviewing different academic databases, we realized that, to the best of our knowledge, the use of the Knowledge Impact Assessment Toolkit has yet to be described within the literature. Therefore, we describe our experience using the toolkit within this manuscript.

## 2 Methods

Working with SCOA and their Caregiver Committee, nurse researchers collaborated on planning the Saskatchewan Caregiver Experience Study and a subsequent knowledge translation webinar for other stakeholders within governmental and nongovernmental organizations in Saskatchewan, as well as members of the public. The webinar activity was our main knowledge translation and mobilization effort for the study. Following the webinar, we assessed our methodology for the Saskatchewan Caregiver Experience Study post hoc using the Knowledge Engagement Impact Assessment Toolkit [10]. Ethical approval was received for the Saskatchewan Caregiver Experience Study from the University of Saskatchewan Behavioural Research Ethics Board (Beh‐ID 3377).

### 2.1 Knowledge Translation Webinar

The knowledge translation webinar, hosted by SCOA [18], was designed as an interactive, community‐facing event aimed at informing caregivers, healthcare professionals, governmental agencies and policymakers and other community stakeholders about the study's results and their implications for caregiver support policy in Saskatchewan. We chose to hold a webinar as they can reach an audience across geographies, and the province of Saskatchewan is spread widely among urban and rural settings. To add to this, the research team was based in Saskatoon (the largest city in the province), while the provincial government is based in Regina (the province′s capital). The objective of the webinar was to share our findings from the Saskatchewan Caregiver Experience study, as well as policy implications and recommendations that we had developed from the study′s findings. Our intended outcome was to garner support for these policy recommendations—most specifically, the recommendations in which SCOA could play an active role in implementing.

As an additional mobilization effort, in advance of the webinar, we mailed coil‐bound packets of our four previous scholarly publications on the Saskatchewan Caregiver Experience Study [8, 11, 13, 14]. The packet included a cover letter from the lead investigator (S.H.) and Executive Director of SCOA (J.G.) providing an overview of the study and an invitation to the webinar. Those interested registered via a Zoom webinar registration page, provided as a QR code on the invitation. During the webinar, we used Padlet [19], an interactive web‐based application for participants to share thoughts and reflections in real‐time on a shared screen by scanning a QR code with their mobile phone or accessing a shortened web link on their desktop computer.

### 2.2 Application of the Knowledge Impact Assessment Toolkit

Herein, we describe our use of the Knowledge Impact Assessment Toolkit. Both the Assessment Matrix and Assessment Portrait were completed highlighting the impact of activities from different phases of the Saskatchewan Caregiver Experience Study. The official usage guide can be requested from the University of Calgary's website: https://research.ucalgary.ca/engage-research/knowledge-engagement/ke-toolkit.

#### 2.2.1 Assessment Matrix

We used the Assessment Matrix to systematically evaluate our project's activities and their relative impact. The Matrix prompts researchers to (1) identify which initiative or activity they are assessing; (2) consider its stage in the knowledge engagement lifecycle (planning, monitoring or impact assessment); (3) review the relevant impact indicators; and (4) assign a score (1–5) for each indicator based on how well the activity addresses that indicator. A weighted average is then automatically calculated in a Microsoft Excel template to provide a total impact score for each activity, classifying it as low, moderate or high impact.

Following the Saskatchewan Caregiver Experience Study, we applied the Assessment Matrix retrospectively (post hoc) to evaluate three key activities of the project: conceptualization, data collection and analysis and knowledge mobilization. The primary author (S.H.) was responsible for assigning the scores for each activity using the Matrix's rating scale (1−5, with 1 being the least mutually beneficial for both researchers and communities and 5 being the most). Upon completing the scoring, the weighted impact scores for each of the three activities were summarized to determine whether they demonstrated low, moderate or high impact. These classifications were then used to guide our interpretation of the project's overall effectiveness in promoting meaningful knowledge engagement and mobilization.

#### 2.2.2 Assessment Portrait

To complement the results from the Assessment Matrix, we incorporated the Assessment Portrait as a qualitative tool to capture the broader narrative and context surrounding our knowledge engagement activities. The Portrait template offers a series of prompting categories and reflective questions designed to illuminate the processes, successes and challenges that might not be fully conveyed through numeric scores alone. We reviewed the prompting categories (reciprocity, externalities/reach, access, partnership and strategic context) and tailored them to align with our project's scope. By organizing these insights within the structure of the Assessment Portrait template, we captured the qualitative dimensions of reciprocity, externalities/reach, access, partnership and strategic context more thoroughly. This approach provided a written narrative to accompany our assessment, enabling us to situate the Matrix scores in the real‐world contexts of partnership development, stakeholder involvement and knowledge exchange processes.

## 3 Findings

### 3.1 Knowledge Translation Webinar

We held our knowledge translation webinar on 28 November 2024. The webinar was attended by 50−60 individuals (attendance varied at different points in the webinar with people signing on and off) from different organizations, such as the provincial Government of Saskatchewan, the Saskatchewan Health Authority, the Alzheimer Society of Saskatchewan, as well as members of the public, some of whom were caregivers. We did not collect demographics on the attendees, as the webinar was an open link that had been circulated to relevant organizations and community members on the SCOA newsletter list. However, we were aware that representatives from the aforementioned organizations attended due to follow‐up communication after the webinar. Representatives from the organizations that attended were relevant systems professionals who have decision‐making responsibilities within Saskatchewan.

The webinar session opened with a brief introduction by the Executive Director of SCOA (J.G.), who welcomed attendees and introduced S.H. as a keynote presenter. S.H. then delivered a detailed presentation of the study's background, methodology, key findings and the policy recommendations derived from caregiver participant responses, developed in tandem with SCOA. The findings were organized geographically, distinguishing between rural and urban caregiver experiences. Each policy recommendation was accompanied by illustrative quotes from study participants and proposed implementation strategies, with special attention given to areas where SCOA could play a leading role with additional support. These policy recommendations and illustrative quotes are presented in Table 1.

**Table 1:** Rural and urban policy recommendations.

Policy recommendation	Policy action	Implementation strategies^a^	Supporting quote(s)^b^
*Rural setting recommendations*			
Strengthen home care workforce	Increase funding for rural home care services to expand their reach and ensure caregivers can rely on professional support in their communities.	Resources are needed for daily tasks—meal prep, hygiene assistance and medication management. Incentivize home care agencies to prioritize rural regions by offering tax rebates or direct subsidies.	“Increasing support for Home care in rural areas, recognizing the challenges of recruitment and retention. Private Home Care is based in larger centres. Often when the caregiver is needing a break, it requires the one requiring care to have to go to a facility (respite), which often initiates a break in routine and causes agitation with someone who has dementia. Sometimes it's just easier to provide all the care rather than change the surroundings, routine, and needs of the person who needs care.” “Lack of Home Care services in rural resulting in those that require care needing to be relocated to larger unfamiliar locations. No support to have a parent move in with family due to lack of staff in rural. Childcare costs are 35–45 dollars a day but to hire a private care provider for older adults is 36/hr.”
Improve access to healthcare and transportation	Strengthen rural healthcare infrastructure and reduce the burden of travel for caregivers.	*Expand telehealth services with specialized support for dementia care, mental health counselling and routine health checkups. Fund mobile medical units to provide lab tests (e.g., phlebotomy, ECG) and consultations in rural communities. Expand and subsidize nonemergency medical transport services for caregivers and their care recipients.	“We live in a small town with no pharmacy, doctor, or care facility. When my mom was in hospital and, later, my dad in care, the number of miles I put on was horrendous. There is no one to call here and every health challenge had to be dealt with an hour away.” “Small towns need to be able to retain [staff in] the healthcare facilities that already exist. It's more than inconvenient to drive 1.5 h for emergency care; it is very hard on the person with dementia.”
Address rural long term care challenges	Improve quality and access to long‐term care in rural communities.	*Quality: Fund training programmes for rural LTC staff to learn more about dementia care, palliative care and so forth. *Quality: Fund organizations that can provide additional support in LTC, such as companionship programmes and activity facilitation. Access: Prioritize enhancement of a centralized LTC placement system to match rural residents with facilities efficiently.	“Improvement in senior accommodations—i.e., nursing homes & levels they accept… the fear of not having an opening in a nursing home in her community & she would have to be placed in a home in another community &/or health region/area.” “My mother fell at home [and was] admitted to a local ER but transferred to a hospital 1 h away due to lack of beds. Spent four days there, transferred to the city for investigation and tests. Transferred to ‘home Hospital' which was 2 h from her home due to lack of beds, three weeks later transferred to the fourth choice on the list for long‐term care. Spent three weeks and then transferred to her final long‐term care facility in her home. She was in 5 institutions, 6 ambulance trips in 7 weeks. Meanwhile, as a caregiver, I had to drive to all these different facilities every two days, at great financial and personal fatigue cost!”
Establish a rural caregiving task force	Establish a provincial task force to address rural caregiving challenges.	*Form an interdisciplinary team of caregivers, HCPs and policymakers to review and recommend changes. *Continue to survey the needs of rural caregivers to identify emerging needs and gaps in services. *Ensure caregiver representation in health policy planning and resource allocation.	“I feel like there needs to be a way for caregivers to know what services are available regarding home care, or other services, and how to go about accessing them. So many times, I feel like we are blindly navigating the healthcare system, and this greatly adds to the stress in our situation.“ “A task force could help navigate and implement changes that consider caregivers' and seniors' needs.”
*Urban setting recommendations*			
Increase affordable respite care services	Expand respite care availability to give caregivers necessary breaks and prevent burnout.	Introduce funded respite care programmes with a sliding fee scale based on income. Establish drop‐in day programmes for care recipients. Create a caregiver relief fund to subsidize private in‐home respite services for low‐income caregivers.	“Having someone provide respite would be a huge help, even if it's just for a short period.” “[I would prioritize] respite care for a few hours so I could leave and know everything was ok.” “Respite or a way to have someone take patient for an outing or companionable visit for enrichment, to allow a caregiver some time for their own needs or wants.”
Expand workplace support for caregivers	Introduce job protection for caregivers of older adults.	Create employment protections for caregivers to ensure flexible work schedules. Promote remote work options where possible for employees with caregiving responsibilities. *Offer subsidies for businesses to implement caregiver employee assistance programmes to offer counselling, navigation resources and so forth.	“There's not enough legislation that allows for long‐term caregivers the understanding and acceptance they need. Familial accommodations in the workplace can be done when young families are having difficulties accessing daycares, etc. When you're caring for an older adult, the patience or tolerance isn't the same.”
Address LTC staffing shortages	Incentivize healthcare professionals to work in urban LTC and home care.	Introduce student loan forgiveness programmes for healthcare workers committing to LTC facilities. Create fast‐track training programmes for healthcare aides specializing in older adult care.	“Long term care staff generally are doing the best they can, short‐staffed often.” “There is a need for affordable, safe, kind, caring, sufficiently staffed facilities.” “I believe there should be more funding of seniors care in the province. I believe there are not enough staff in these homes to properly care for the elderly.”
Advocacy and public awareness campaigns	Raise awareness about caregiving challenges and available supports.	*Fund public education campaigns about caregiving resources, including workshops and seminars. *Support SCOA and SCISC in recognizing caregiver contributions during the annual provincial caregiver appreciation week in April.	“Caregivers require the respect and support of the healthcare system. By supporting caregivers, we are improving patient care.” “More advocacy and someone to explain and take the time to assist seniors to better understand medical appointments, treatments and medications.” “There needs to be clear and standard ‘rules' about including and informing family or chosen support persons. [We] so often get lost or misunderstood. We should not have to fight to be part of our loved one's care team.”
Improve navigation of health and social systems	Centralize caregiving assistance to access information and resources.	*Support a ‘one‐stop caregiving shop' to provide guidance on caregiving activities (e.g., Caregiver navigators at the Saskatoon Caregiver Information and Support Centre) *Caregiving support help line (already in existence through SCOA).	“There are a lot of services but you're on your own for coordinating it all. The geriatric assessment at City Hospital was good – but they just give you a long list of ‘here, this is what you need to do, bye‐bye!' Help but not particularly helpful – it's great to have the input but it's getting it all done that's challenging and exhausting.” “Finding resources for them and knowing how to navigate the systems… Always digging for what is needed. Haven't found anything easy!”

^a^ Strategies with an asterisk (*) are those we highlighted as SCOA being well‐positioned to assist with implementing.

^b^ Quote from the Saskatchewan Caregiver Experience Study to support the policy recommendation. Methodology of the study is reported elsewhere (Hall et al. 2024). Quotes are from urban or rural caregivers, respectively.

Following the presentation by S.H., the session transitioned to a reflection and response segment. A representative from SCOA's Caregiver Information and Support Center (J.W.), provided remarks that contextualized the study's findings within broader provincial ageing and caregiving trends. J.W. emphasized the urgency of systemic change and encouraged collective action among attendees. The event concluded with an invitation for participants to contribute their perspectives via the Padlet board and to access related resources, including the published journal articles and SCOA's contact information for further engagement. Unfortunately, the Padlet app was not used very actively during the webinar, with only a few comments of appreciation and encouragement posted.

### 3.2 Assessment Matrix

**Figure 1:**
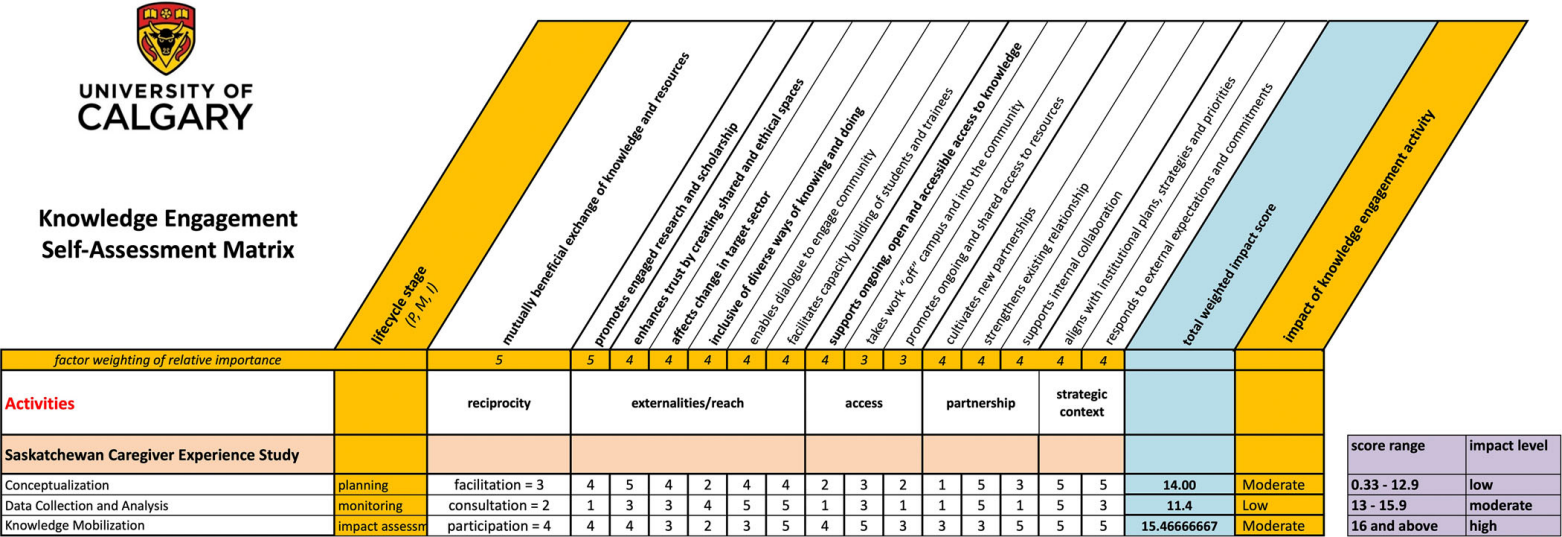
Knowledge Engagement Self‐Assessment Matrix [10] completed post hoc for the Saskatchewan Caregiver Experience Study.

Following the webinar, we used the Assessment Matrix Excel spreadsheet template to evaluate three key activities from the Saskatchewan Caregiver Experience Study (conceptualization, data collection and analysis and knowledge mobilization) across various impact indicators. Figure 1 presents our assessment matrix using the Excel spreadsheet template from the University of Calgary [10]. Each indicator was rated on a scale of 1−5, with higher scores reflecting greater responsiveness to that impact measure and weighted to emphasize its relative importance (Weighted Impact Score). The spreadsheet automatically generated a total weighted impact score for each activity and classified the overall impact as low, moderate or high based on predefined scoring thresholds.

The conceptualization phase achieved a total weighted impact score in the moderate range. Higher scores on indicators such as collaboration (reciprocity) and strategic alignment suggested strong early‐stage engagement with SCOA, our community partner. However, slightly lower values in certain complementary indicators (e.g., ongoing access to resources) tempered the overall score. In contrast, the data collection and analysis phase yielded a total weighted impact score in the lower range, resulting in a classification of low impact. While there were robust elements of community consultation, certain aspects received lower ratings in the Matrix, such as multi‐directional knowledge exchange and capacity building. This outcome highlighted areas where deeper or more consistent engagement with SCOA might have enhanced the perceived impact. Finally, the knowledge mobilization phase (i.e., the webinar) scored in the moderate impact range. The spreadsheet results suggest that outreach and dissemination efforts successfully engaged target audiences and facilitated collaboration, but some indicators (e.g., externalities that extend beyond the immediate partnership) did not reach the highest possible scores. This result is due to the fact that policy recommendations and implementation strategies have yet to be enacted, to our knowledge.

By comparing the total weighted impact scores across these three activities, we gained insights into which dimensions of knowledge engagement were most robust and where improvements could be made. This classification informed our subsequent interpretations about how effectively the project as a whole advanced meaningful community partnerships, reciprocal knowledge sharing and potential longer‐term benefits for caregivers in Saskatchewan.

### 3.3 Assessment Portrait

Although the Weighted Impact Score from the Assessment Matrix provided a concise summary of each activity's performance, supplementing it with qualitative reflections ensured a fuller picture of our knowledge engagement practices. Herein, we detail our reflections and ‘assessment story’ [10].

#### 3.3.1 Reflections

##### 3.3.1.1 On Weighted Impact Score

Achieving a numeric snapshot of impact helped clarify how each of our knowledge engagement activities (conceptualization, data collection and analysis and knowledge mobilization) aligned with the principles of reciprocity, externalities, access and partnership, as well as strategic context. The conceptualization and knowledge mobilization phases were rated moderate, suggesting solid collaborative efforts and multi‐directional knowledge flows, whereas data collection and analysis scored lower, highlighting an opportunity for deeper community integration at that stage. By linking these numeric scores to broader considerations, such as relationship building, trust and long‐term sustainability, we were able to see how and where certain phases excelled and where additional support or realignment may be warranted.

##### 3.3.1.2 On Assessing Impact

Responsive communication, the value of open dialogue and the importance of adapting tools (e.g., tools to address information needs) to meet local needs are significant strategies to be implemented to ensure impact. The Assessment Matrix served as a reflection tool for three intervals of the Saskatchewan Caregiver Experience Study, which prompted us to consider who benefited, how information flowed and whether community voices were sufficiently represented. Findings from the Saskatchewan Caregiver Experience Study revealed that many of the challenges caregivers face require governmental action, including increased funding, legislative reform and interdepartmental collaboration. Even high‐impact engagement at the community level can stall if policies remain unchanged or funding is inconsistent, particularly in rural areas with fewer available supports. The continued push for policy recognition and dedicated funding not only aligns with caregivers' evolving needs but also ensures that knowledge engagement outcomes remain viable and impactful beyond the lifespan of any single project.

##### 3.3.1.3 On the Knowledge Engagement Journey

Embracing a knowledge engagement journey mindset allowed us to continuously refine and adapt our approach in our partnership with SCOA. A longitudinal perspective of ‘engagement as a journey’ incites reflection to respond to needs, such as creating the plain‐language summaries of key insights for community newsletters or advocating for new community‐based government‐funded services through our knowledge translation webinar and ultimately fosters a sustained commitment to reciprocal knowledge exchange beyond the study′s formal conclusion.

#### 3.3.2 Assessment Story

##### 3.3.2.1 Reciprocity

In the conceptualization phase, we ranked our reciprocity as ‘3 = facilitation’. Facilitation is where the researchers and community organizations support each other to generate new knowledge. Our collaboration with SCOA had already been longstanding, and so we worked together through planning meetings to decide the approach to conducting the Saskatchewan Caregiver Experience Study. In the data collection and analysis phase, we ranked our reciprocity as ‘2 = consultation’. This ranking was selected, as it reflects that external (community) insights and feedback are sought on the knowledge is being generated by researchers. The data collection and analysis were carried out solely by the research team, and then insights and feedback were sought on the findings from SCOA. Lastly, our mobilization phase, which involved our knowledge translation webinar, ranked ‘4 = participation’. This phase is where researchers and community organizations both contribute expertise and ideas to generate new knowledge, leading to increased understanding and mutual benefit.

##### 3.3.2.2 Externalities and Reach

This facet of the matrix was rated highly in the conceptualization and mobilization phases, and lower in data collection and analysis. Data collection and analysis ranked lower due to the ‘behind the scenes’ coding of data, in which SCOA was not directly involved. In the mobilization phase, we collaborated on our webinar, promoting engaged research and scholarship. This activity sought to affect change in the targeted sectors (governmental and health systems) and enabled dialogue to engage with the caregiving community and other knowledge users. We have also shared our results via local and national conferences (e.g., Canadian Association on Gerontology in Edmonton, AB; Canadian Geriatrics Society in Vancouver, BC; Encircling Care Summit and Saskatchewan Health Research Showcase in Saskatoon, SK), in newsletters (e.g., SCOA monthly newsletter; Saskatchewan Red Cross newsletter) and media engagements (e.g., CBC Saskatchewan Radio). As such, our research influenced an expanding audience, from individual caregivers to larger organizations and health authorities.

##### 3.3.2.3 Access

For the access domain, we felt, in general, our activities in our study ranked moderately. Higher‐end scores were ranked in the category where research is taken ‘off campus’ and into the community, due to our partnership with SCOA in crafting our policy recommendations. Lower‐end scores were ranked in supporting ongoing, open and accessible access to knowledge, as the data collection and analysis happened ‘behind the scenes’. Lastly, the shared access to resources ranked lower, as SCOA was only shared published and complete data, rather than raw collected data before analysis.

##### 3.3.2.4 Partnership

The University of Saskatchewan's College of Nursing and SCOA have had a long‐standing collaborative relationship. Therefore, in the conceptualization and data collection and analysis phases, ‘cultivating new partnerships’ ranked lower, but ‘strengthening existing relationships’ ranked higher. However, in the mobilization phase, we ranked the new partnership facet in the middle range, due to our engagement with other and new knowledge users.

##### 3.3.2.5 Strategic Context

Overall, our knowledge engagement aligned with local, provincial and national strategies to support older adults to age in place while reducing caregiver burden. Provincially, findings and recommendations from our assessments are targeted to resonate with Saskatchewan's Ministry of Health regarding community care and caregiver support. At the same time, SCOA has incorporated these insights into their strategic planning around resource allocation and programming. The University of Saskatchewan's College of Nursing places emphasis on community‐engaged scholarship, which is reflected in our approach, whereby researchers and community stakeholders cocreate evidence‐driven, user‐focused tools. Additionally, our ongoing dissemination efforts advance broader national discussions about the essential role of caregivers, informing policy advocacy and strategic planning in health and social services across Canada.

## 4 Discussion

Our overarching reciprocity story with SCOA revolves around a two‐way exchange of ideas and resources with caregivers, community partners and academic teams. In early caregiver research work with SCOA in January 2020, we conducted focus groups and an environmental scan ensuring caregivers' voices shaped priorities for new interventions [20]. Likewise, our scoping review on caregiver support [2] fed into the development of the Caregiver Orientation Guide [16]. This cycle of collaborative planning, data collection and results‐sharing has fostered ongoing dialogue in which our research influenced SCOA project directions and benefited from practical support tools. Our work has helped SCOA prioritize making supportive information and tools available to caregivers of older adults, many of whom have previously reported feeling isolated or unaware of existing services [20]. The Caregiver Orientation Guide [16] was posted on SCOA's website and distributed through community networks, offering step‐by‐step assistance for topics such as navigating healthcare systems, managing finances and performing basic care tasks. Additionally, results from the Saskatchewan Caregiver Experience Study highlighted unique barriers for rural caregivers (e.g., limited local respite options), prompting the vision of expanding the SCISC provincially. This emphasis on user‐friendly, on‐demand formats (e.g., downloadable PDFs, as well as printed toolkits) ensures that knowledge is accessible to those in need.

### 4.1 Implications for Public Health

As the population ages, caregivers increasingly take on essential roles in sustaining the well‐being of older adults in the community [1]. When caregivers are unsupported, or when they lack essential resources such as respite care, mental health services and financial assistance, both caregivers and care recipients can experience poorer health outcomes [21]. Thus, investing in caregiver supports at the population level is a strategic approach to reduce system‐wide burdens and foster healthier communities [21]. Informal caregiving intersects with multiple social determinants of health, including economic stability, social support networks and access to healthcare and community services [21, 22, 23]. Caregivers often report financial hardships, workplace challenges and higher rates of stress, anxiety and depression [21, 22]. These factors, in turn, can diminish caregivers' capacity to care for older adults effectively. Within a social determinants framework, policies that ensure caregivers have access to fair workplace policies (e.g., paid leave and flexible scheduling), financial aid and educational programmes can help mitigate the cascading negative effects on caregivers' well‐being [22, 24]. A public health lens calls for intersectoral collaboration among healthcare, social services and employment sectors to provide holistic supports that strengthen caregivers' resilience and capacity [22, 23].

### 4.2 Strengths and Limitations

A strength of this initiative lies in its focus on knowledge translation and mobilization. The Knowledge Engagement Impact Assessment Toolkit [10] offered a structured method to evaluate how research findings were moved into real‐world settings. Systematic, evidence‐based approaches to knowledge translation are essential to reducing the gap between what is known (e.g., effective caregiver interventions) and what is done (e.g., policies implemented, services provided). By engaging with SCOA and convening a webinar with stakeholders from government, healthcare and the community, this study translated findings into proposed policy changes and programme adaptations. This style of knowledge mobilization helps ensure that the lived experiences of caregivers directly inform system‐level solutions.

Despite the strengths of this study, limitations also warrant consideration. Although the Knowledge Engagement Impact Assessment Toolkit provided a structured framework to evaluate engagement processes, we applied it post hoc rather than prospectively. This timing may have constrained our ability to adapt engagement strategies in real time. As well, the moderate impact ratings from the conceptualization and mobilization phases, contrasted with the lower impact score in data collection and analysis, highlight that deeper collaboration with community partners at each research stage could have yielded a more robust reciprocal exchange. Furthermore, caregivers who attended the event were given the opportunity to reflect on the findings and knowledge translation process at the webinar via the Padlet application; however, as previously described, there was inadequate uptake of the app during the webinar. In the future, a more in‐depth description on how interactive apps such as Padlet can be engaged with will be employed. These limitations underscore the need for ongoing, adaptive research and engagement strategies that proactively deepen community collaboration and employ diverse knowledge mobilization methods.

## 5 Conclusion

Findings from the Saskatchewan Caregiver Experience Study [8, 11, 13, 14] highlight informal and unpaid caregiving as a critical component of healthy ageing strategies. By applying the Knowledge Engagement Impact Assessment Toolkit [10], we gained a systematic, reflective framework to evaluate our engagement processes, from conceptualizing research questions alongside community partners to mobilizing findings through webinars and policy dialogues. This structured approach underscored that knowledge translation and mobilization efforts require both a quantitative measure of reach and a qualitative understanding of reciprocities, partnership dynamics and real‐world application.

The moderate impact scores assigned to the conceptualization and mobilization phases suggest meaningful, multi‐directional exchange between researchers, community organization and policymakers. However, the relatively lower score for data collection and analysis points to opportunities for deeper cocreation of knowledge, particularly in refining data collection techniques and improving feedback loops with community stakeholders. A public health lens is especially relevant here, as unpaid caregiving intersects with the social determinants of health, influences healthcare utilization and substantially affects caregiver well‐being. Effectively translating evidence into policy and practice can reduce caregiver burden, promote mental health and foster sustainable ageing‐in‐place solutions.

Building on the relationships fostered and insights gathered from the Saskatchewan Caregiver Experience Study, we anticipate a lasting collaborative bond with SCOA. This partnership has laid the groundwork for ongoing, reciprocal exchange, fostering future community‐driven projects and practical caregiver supports. Beyond strengthening partnerships, the Saskatchewan Caregiver Experience Study's robust evidence base creates a strong platform for advocating for wider policy change and directing further investment in caregiver initiatives.

## Ethics Statement

This study received ethical approval from the University of Saskatchewan's Behavioural Research Ethics Board (Beh‐3377).

## Conflicts of Interest

The authors declare no conflicts of interest.

## Data Availability

The data that support the findings of this study are available from the corresponding author upon reasonable request. No additional data is available.
